# Does the McRoberts’ manoeuvre need to start with thigh abduction? An innovative biomechanical study

**DOI:** 10.1186/s12884-020-02952-6

**Published:** 2020-05-04

**Authors:** David Desseauve, Laetitia Fradet, Robert B. Gherman, Yosra Cherni, Bertrand Gachon, Fabrice Pierre

**Affiliations:** 1 Department of Obstetrics and Gynecology and Reproductive Medicine, University Hospital of Poitiers, Poitiers, France; 2 Pprime Institute, CNRS UPR 3346, Axis RoBioSS, University of Poitiers, Poitiers, France; 3 Department of Obstetrics and Gynecology, Centre Hospitalier Universitaire Vaudois (CHUV), 1011 Lausanne, Switzerland; 4 Department of OB/GYN, Division of Maternal/ Fetal Medicine, Wellspan Medical Center, York, PA USA

**Keywords:** Shoulder dystocia, McRoberts manoeuvre, Biomechanical, Motion capture system, Lumbar curve

## Abstract

**Background:**

Guidelines and description about the achievement of the McRoberts manoeuvre are discordant, particularly concerning the need for abduction before the beginning of the manoeuvre. We sought to compare the biomechanical efficiency of the McRoberts’ manoeuvre, with and without thigh abduction.

**Methods:**

In a postural comparative study, twenty-three gravidas > 32 weeks of gestational age and not in labour were assessed during three repetitions of two McRoberts’ manoeuvre that differed in terms of starting position. For the (i) McRoberts, the legs were initially placed in stirrups; for the (m) McRoberts, the legs were resting on the bed, with thighs in wide abduction. For each manoeuvre, flexion of the plane of the external conjugate of the pelvis on the spine (ANGce), hip flexion and abduction, were assessed using an optoelectronic motion capture system. Lumbar curve were assessed with Epionics Spine® system. Temporal parameters including movement duration or acceleration of the external conjugate were also computed. All values ​​obtained for the two types of manoeuvres were compared using a Wilcoxon matched-pairs signed-ranks test. The significance level was defined as *p* < 0.05.

**Results:**

The starting position of McRoberts’ otherwise had no effect on the maximum ANGce (*p* = 0.199), the minimal lordosis of the lumbar curve (*p* = 0.474), or the maximal hip flexion (*p* = 0.057). The other parameters were not statistically different according to the starting position (*p* > 0.005).

**Conclusion:**

Regardless of the starting position, the McRoberts’ manoeuvre allows ascension of the pubic symphysis and reduction of the lumbar lordosis. This results imply that the McRoberts’ manoeuvre could be performed with the legs initially placed in the stirrups.

## Background

The McRoberts’ manoeuvre was initially described as a “communication in brief” in 1983 by Gonick et al. [1]. In the ensuing years, it has become the first-line treatment for the alleviation of shoulder dystocia based upon its simplicity of application and relatively high success rates [2]. In a retrospective review of 250 shoulder dystocia cases that occurred between 1991 and 1994 at Los Angeles County-University of Southern California, the McRoberts’ manoeuvre alone was found to have a success rate of 42%. More than half (54.2%) of the shoulder dystocias were resolved with the combination of McRoberts’, suprapubic pressure, and/or proctoepisiotomy [3]. The mechanism of action of the McRobert’s manoeuvre perform a rapidly marked anterior rotation of the pubic symphysis and by flattening the sacrum. This manoeuvre might allow for anterior foetal shoulder elevation, pushing of the posterior foetal shoulder over the sacrum, and brings the pelvic inlet perpendicular to the maximum expulsive forces [3].

Gonik’s initial description simply stated that, “the patient’s legs were removed from the stirrups and sharply flexed against her abdomen.” [1]. However, the initial position of the parturient was not specified in this case report. Furthemore, there is no current consensus on other parameters concerning the position of the woman before and during the manoeuvre in terms of flexion or abduction of the thighs, nor other parts of the body (position of the upper limbs, head inclination) [4, 5]. Consequently in clinical practice, there is a wide variation of the McRobert’s manoeuver technique between hospitals.

The majority of the parturients can undergo the McRobert’s manoeuvre as described either with help of the health-care providers in labor-ward. Women that could experience difficulties in performing this manoeuvre include women with morbidities associated with the specific rapid movement of the lower limbs and hips and assuming a dorsal lithotomy position: obese patients, lower-limb and pelvic fractures, spinal-cord injuries, neuromuscular disorders, osteoarthritis, rheumatoid arthritis or other severe degenerative joint disorders .

In this study, we sought to compare the biomechanical efficiency of the McRoberts’ manoeuvre depending on the initial position/abduction degree of the thighs, namely, with or without abduction before manoeuvre accomplishment.

## Methods

In this prospective comparative study, eligible participants were gravidas older than 18 years and > 32 weeks’ gestation, based on dating criteria of last menstrual period or first trimester ultrasound. Exclusion criteria included a body mass index > 40, a maternal medical condition that prevented maternal hip hyperflexion, inflammatory joint diseases, or joint hypermobility syndrome, such as Marfan’s syndrome. The study protocol was approved by the Ethics Committee of Poitiers Hospital (Comité de Protection des Personnes: 2013–1203-42) and by the French National Agency of Drug Safety (Agence Nationale de Sécurité du Médicament: B131–460-22). All women provided written informed consent. No financial incentive was offered for participation. This biomechanical study took place in an experimental setting (i.e., not during labour). Medical students in their 4th year of training, who had undergone pre-study briefing and training, performed the manoeuvres. A birthing bed (Maquet®) was used with an angle of inclination of the headboard of 30° (typical placement of the parturient at Poitiers’s hospital).

A full protocol description about this innovative methodology is available in a recent publication [6]. A traditional three-dimensional motion analysis was performed to analyse the position of the markers in space. It was based on an optoelectronic motion capture system consisting of 12 infrared cameras cadenced at 100 Hz (VICON, Oxford Metrics, UK). Thirty-three reflective markers were affixed using double-sided tape on anatomical landmarks according to an adapted version of the Helen Hayes’s marker set [7]. To assess the position of the pelvis, we placed additional markers on the pelvis. An antenna fitted with three markers was positioned on the top of each iliac crest to provide a technical coordinate system, allowing the reconstruction of the pelvic markers if they were to be hidden during the experimentation. Marker trajectories were low-pass filtered using a double-pass Butterworth filter with a cut-off frequency of 10 Hz.

The lumbar curve was assessed by measuring the lordosis according to the Epionics SPINE system (Epionics Medical GmbH, Potsdam, Germany). This system consists of two flexible sensor strips that use strain gauge sensors located alongside flexible circuit board strips. The positioning of the system is standardized. According to this measure, a lordosis of 0° corresponds to a back perfectly flattened. The data acquisition (50 Hz) was transmitted in real time via Bluetooth to a local PC [6].

**Fig. 1:**
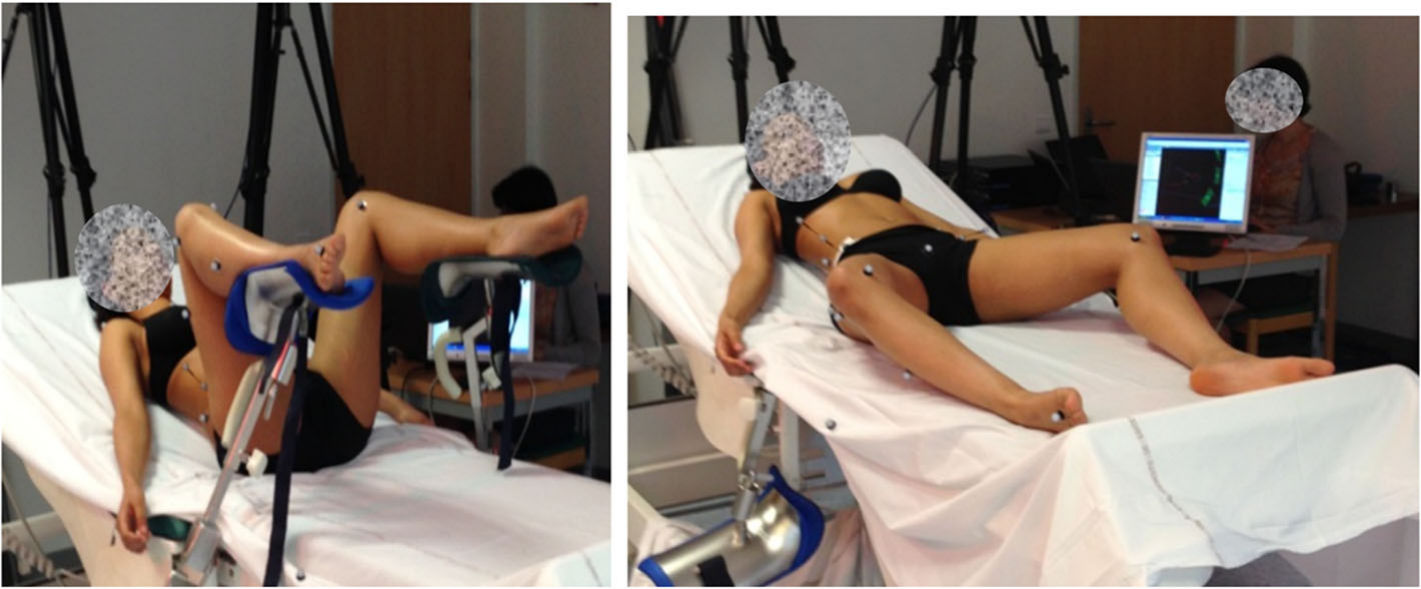
Illustration of starting position for “initial” (*iMcRoberts*) and “modified” (*mMcRoberts*) McRoberts manoeuvre


Subjects were positioned in the lithotomy position, with the thighs lying on the stirrups, with a flexion of 90°. Two medical students performed the McRoberts manoeuvre as initially described by Gonik [1] ((i) McRoberts). Afterward, the subjects were placed with their legs outside the stirrups, with the feet lying on the bed and the thighs in a neutral position in terms of flexion but with a maximal abduction and femoral external rotation. The same students again applied simultaneous maximal flexion of the thighs at the request of the investigator. This modified McRoberts’ manoeuvre was referred to as (m)McRoberts. The sequence (i) McRoberts then (m) McRoberts, which corresponds in fact to two different starting positions (Fig. 1), was carried out 3 times for each subject. The medical students used to employ the manoeuvres were different for each patient, in order to avoid any training effect. Each manoeuver was performed under the supervision of a senior obstetrician (DD) to check the appropriate achievement of the manoeuvre. Examples of the manoeuvres after reconstruction are available in supplementary files added to this article.

**Additional file 2.** MOSM2 as mMcRoberts.


**Fig. 2:**
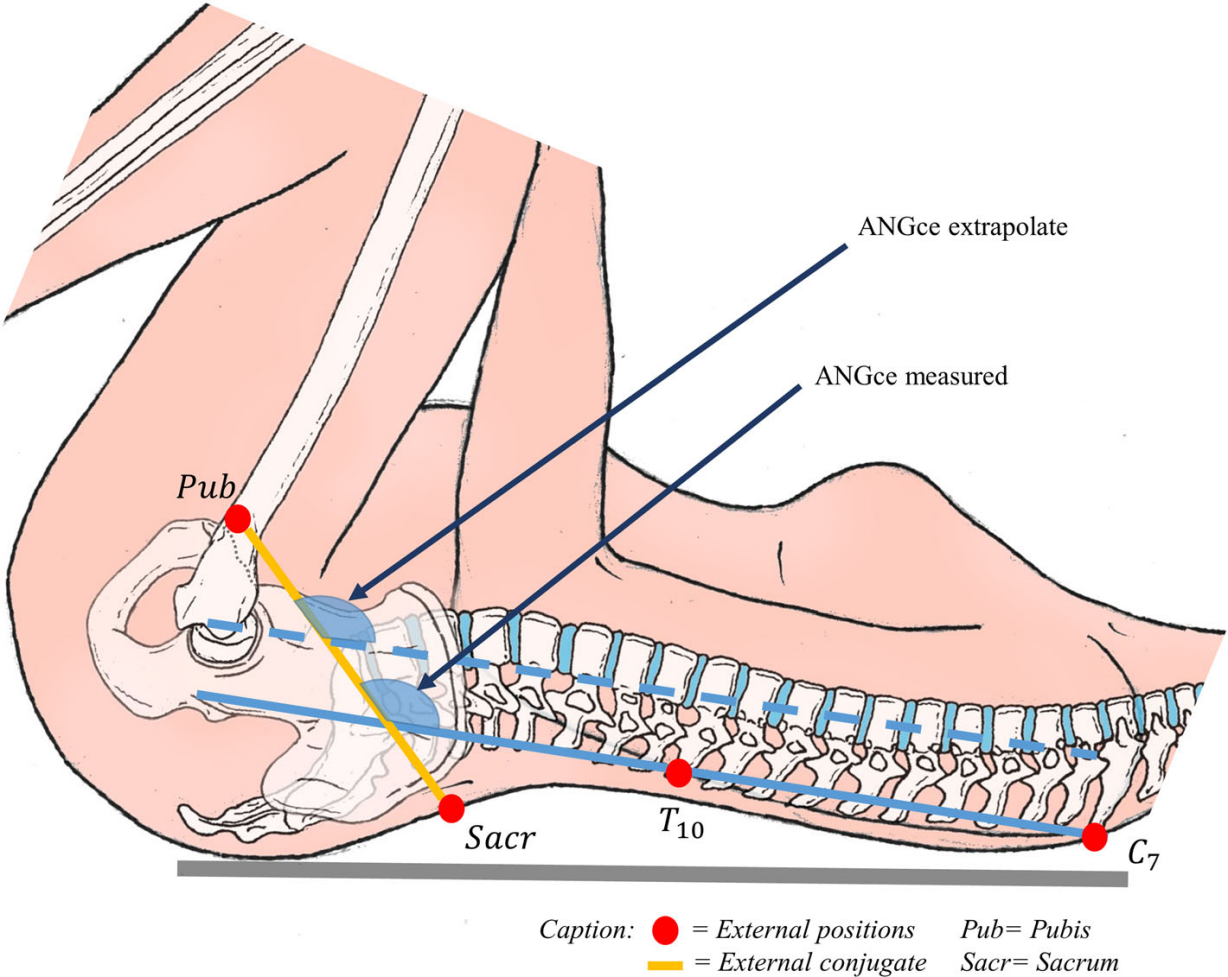
Definition of ANGce and external conjugate

A custom Matlab code (MathWorks Inc., Natick, MA) was used to merge data from Epionics and optoelectronic systems and to extract the required data. We defined a plane following the external conjugate diameter using the two markers placed on the posterosuperior iliac spines and the marker placed on the superior edge of the pubic symphysis. The hip joints angles (flexion and abduction) were obtained as defined by the conventional gait model [7]. The flexion of the plane of the external conjugate on the spine (***ANGce***) was defined in the sagittal plane as the angle between the external conjugate and the line defined by the markers placed on the 7th cervical and the 10th thoracic vertebrae (Fig. 2). The lumbar curvature was measured during the manoeuvre for each subject. For each angle, the initial and maximum values ​​were noted. To provide a better description of the manoeuvre, the maximum angular acceleration of the flexion of the external conjugate on the spine. The angular acceleration was obtained by a double differentiation (numerical method by differentiation decentred on the right) of the angle ***ANGce***. The total duration of the movement was also calculated. The beginning of the movement was defined as the time at which the angular velocity of the thigh flexion exceeded 5% of the maximal angular velocity reached during the manoeuvre. The end of the movement was defined as the moment when the angular velocity of a segment (thigh or pelvis) became less than 5% of the maximal angular velocity reached during the manoeuvre. The beginning of the external conjugate diameter flexion, the maximum flexion acceleration were determined. Durations were expressed relative to the moment of the beginning of the manoeuvre. The values obtained during each manoeuvre were recorded and averaged over the three repetitions of each manoeuvre.

Based on the previous results of Gherman et al. [3], we considered as significant, a 4° (5%) increasing of ANGce. With this hypothesis, 22 participants are necessary with of power of 90% and a risk of type I error of 5%. All values ​​obtained for the two types of manoeuvres ((i) McRoberts and (m)McRoberts) were compared using a Wilcoxon matched-pairs signed-ranks test. The significance level was defined as *p* < 0.05.

## Results

None of the 23 participants withdrew after giving informed consent. The mean age of the participants was 32.7 (SD 2.9) years, and the mean of the term at the inclusion was 34.2 (SD 3.1, Min 32 Max 40) weeks’ gestational age. The mean body mass index was 26.3 (SD 3.2) kg/m^− 2^. Six participants (26%) were primigravid.

According to Table 1, the starting positions were very different between the two types of manoeuvres, with (i) McRoberts demonstrating logically higher initial hip flexion (*p* < 10^− 5^) and lower initial abduction (*p* = 0.001) than (m)McRoberts. ***ANGce*** was lower (*p* = 0.009) for the (m) McRoberts than for the (i) McRoberts manoeuvre. During the manoeuvre, the starting position did not affect the maximum ***ANGce*** angle (*p* = 0.199), the minimal lordosis of the lumbar curve (*p* = 0.474), or the maximal hip flexion (*p* = 0.057). A slightly higher maximal abduction for the (m) McRoberts than for the (i) McRoberts (*p* < 0.001) was noticed during the manoeuvre.

**Table 1:** Data presented as mean + standard error of the mean

***N = 23***	***iMcRoberts***: $\overline{\boldsymbol{X}}$ (SD)	***mMcRoberts***: $\overline{\boldsymbol{X}}$ (SD)	***p***
**Initial values**			
Hip flexion (°)	85 [8]	34 [17]	< 0.001
Hip abduction (°)	38 [4]	48 [5]	0.001
ANGce (°)	–7 [10]	−11 [9]	0.009
Lumbar curve (°)	−7 [5]	−8 [6]	0.072
**Maximal values**			
Hip flexion (°)	120 [8]	115 [13]	0.057
Hip abduction (°)	35 [6]	43 [8]	0.000
ANGce (°)	11 [9]	10 [9]	0.199
Lumbar curve (°)	2 [12.2]	−0.2 [5.9]	0.474
**Dynamics and temporal parameters**			
Pelvis angular acceleration (rad.s^− 2^)	0.23 [0.09]	0.27 [0.11]	0.095
Movement duration (s)	1.40 [0.25]	1.37 [0.21]	0.518
Time of the initial pelvis movement (s)	1.03 [0.30]	1.06 [0.15]	0.397
Time of the maximal pelvis acceleration (s)	0.92 [0.25]	0.82 [0.21]	0.150

The maximum acceleration of the pubic symphysis was not significantly affected by the starting position (*p* > 0.05). The duration of the motion, the time of onset of movement of the external conjugate diameter, and the peak of the external conjugate acceleration were not statistically different according to the starting position (*p* > 0.005).

## Discussion

In this biomechanical study, we confirmed that the McRoberts’ manoeuvre, regardless of the starting position, allows ascension of the pubic symphysis and reduction of the lumbar lordosis.

In the initial article that described the McRoberts’ manoeuvre, Gonik specified that the thighs of the patient must be flexed against her abdomen [1]. However, it was also mentioned that the woman’s feet must be released from the stirrups before performing the manoeuvre, which implies that the manoeuvre was performed from a conventional lithotomy position. We recognize that the starting position used in this study (a wide abduction and with the leg on the delivery table) may rarely be adopted in certain maternity wards, in particular in American maternity wards. Indeed, for the majority of the parturients in France, the stirrups are not removed before McRoberts’ is performed, starting from a dorsal lithotomy position. According to our results, initial abduction does not improve the manoeuvre and the effectiveness of the McRoberts manoeuvre does not seem to be dependent on the initial flexion and abduction of the thighs. To explain this, we propose that, in fact, there is “nothing more to gain,” as the manoeuvre consisting of hip hyperflexion already provokes maximum pelvis movement.

The major issue in the management of shoulder dystocia is to prevent foetal trauma and neurologic damage. The first recommended manoeuvre performed is the McRoberts manoeuvre, which should be effective for alleviating the anterior shoulder. Forthcoming research on optimization of this well-known manoeuvre involves defining the easiest movement, particularly in terms of its control. The challenge will be to reduce the force that is applied on the shoulder by the pubic symphysis in order to diminish the stretching of the brachial nervous fibres. Modelling should help us in this task and help us to better understand the mechanism of the McRoberts manoeuvre. Further biomechanical modelling studies could elucidate the role of each action.

According to a literature search using MEDLINE and PUBMED, covering 1966 to February 2019 (MESH term: “McRoberts”, and/or “biomechanical”, and/or “mechanical”, and/or “shoulder dystocia”), we believe that this study represents the first observation assessing the effect of hip abduction during the McRoberts manoeuvre. The unique strength of our analysis is the highly precise quantification and characterization of pelvic tilt and lordosis during the McRoberts manoeuvre using a motion analysis system. A single prior biomechanical study involved anterior-posterior and lateral X-rays taken with gravidas in the dorsal lithotomy position and after application of the McRoberts manoeuvre [8].

Our study is further limited because our motion capture methodology was unable to assess the size of the pelvic inlet and dimensions of the pelvis outlet, as previously reported by Gherman [8]. The notion that abduction affects the pelvis size dates to back to 1969, when Russell noticed that “if the thighs are flexed and abducted the femora act as lever on the innominate bones to open the bony outlet” [9]. In 1899, at the 3rd International Congress of Gynecology and Obstetrics, Bonnaire and Bue reported that the accentuated lithotomy position increased the bisischiatic diameter by as much as 16–18 mm and slightly reduced the conjugate diameter. A modification of pelvic size during McRoberts’ manoeuvre, with a wide abduction of the thighs, could modulate our conclusion about the impact of thigh abduction. Nevertheless, the change in pelvic size is tenuous and limited to some millimetres, and the potential effect of additional abduction must be tempered [8]. Therefore, additional studies are needed to measure the size of the pelvis during the McRoberts manoeuvre performed with thighs in maximum abduction.

## Conclusion

Inducing a McRoberts manoeuvre with a wide abduction does not improve the biomechanical effect. A starting position with the legs in the stirrups, followed by a movement of hyperflexion of the thighs, is the most efficient action when shoulder dystocia occurs.

## Supplementary information

**Additional file 1.** MOSM1 could be identify as iMcRoberts.

## Data Availability

The datasets used during the current study are available from the corresponding author on reasonable request.

## Abbreviations

*ANGce*: External conjugate of the pelvis on the spine
*(i) McRoberts*: McRoberts’ manoeuvre as initially described by Gonik (1), with the legs initially placed in stirrups
*(m) McRoberts*: Modified McRoberts’ manoeuvre, the legs were resting on the bed, with thighs in wide abduction
